# Interesting Images in Obstetrics and Gynecology

**DOI:** 10.3390/diagnostics14141558

**Published:** 2024-07-18

**Authors:** Nicolae Gică

**Affiliations:** 1 Gynecology Department, Faculty of Medicine, Carol Davila University of Medicine and Pharmacy, 020021 Bucharest, Romania; gica.nicolae@umfcd.ro; 2 Filantropia Clinical Hospital of Obstetrics and Gynecology, 011171 Bucharest, Romania

Dear Readers,

I am writing this letter to express my appreciation for the authors who published insightful articles in this Special Issue, “Interesting Images in Obstetrics and Gynecology”. As an academic and professional actively involved in this discipline, I find great significance in visual representations that enhance our understanding and care of women’s health. 

This Special Issue, part of the journal *Diagnostics*, brings to our attention innovative and educational information in the fields of obstetrics and gynecology across various images that provide valuable insights into the complexities of human reproduction, gynecologic oncology, high-risk pregnancy, genital malformation, and women’s health.

First of all, the published manuscripts contain important images that serve as important diagnostic tools in antenatal monitoring, gynecology, and high-risk pregnancy using advanced imaging technologies. Ovarian pathology is described in-depth by Mongelli et al. and Gică et al., using insightful images related to very rare cases of the coexistence of neoplastic Müllerian epithelial and sex cord-stromal elements and a pictorial essay about ovarian germ cell tumors [1,2]. Scientific and technological innovation has revolutionized the way we approach various conditions to achieve a more accurate diagnosis and appropriate treatment, enhancing our ability to provide personalized and effective management tailored to each patient.

Moreover, the published articles offer incredible insights and images of very rare pathologies, fetal abnormalities, and life-threatening conditions in high-risk pregnancies, as shown in Bachmann et al., Gireada et al., Tantipalakorn et al. and Pongsatha et al.’s manuscripts [3,4,5,6].

Furthermore, illustrations of surgical procedures in obstetrics and gynecology provide valuable insights into the intricate techniques employed by gynecologists to address perspectives and advanced knowledge. These images serve as educational tools for both aspiring medical professionals and patients. The challenges addressed align perfectly with this Special Issue’s aims. All international submitting authors share detailed illustrated cases, establishing new directions in the fields of obstetrics and gynecology and the challenges associated with mother and fetus healthcare.

In conclusion, I invite you to read the articles published in our Special Issue, which will undoubtedly enrich and further the quality of care in obstetrics and gynecology. I want to thank you for your dedication in this important field, and I look forward to witnessing a continued commitment to research in obstetrics and gynecology, advancing education and patient care through the findings of this Special Issue. 
